# The Link Between the Microbiota and HER2+ Breast Cancer: The New Challenge of Precision Medicine

**DOI:** 10.3389/fonc.2022.947188

**Published:** 2022-07-13

**Authors:** Martina Di Modica, Valeria Arlotta, Lucia Sfondrini, Elda Tagliabue, Tiziana Triulzi

**Affiliations:** ^1^ Molecular Targeting Unit, Department of Research, Fondazione Istituto di Ricovero e Cura a Carattere Scientifico (IRCCS) Istituto Nazionale dei Tumori, Milan, Italy; ^2^ Dipartimento di Scienze Biomediche per la Salute, Università degli Studi di Milano, Milan, Italy

**Keywords:** microbiota, HER2/neu, targeted therapy, breast cancer, precision medicine

## Abstract

The microbiota is emerging as a key player in cancer due to its involvement in several host physiological functions, including digestion, development of the immune system, and modulation of endocrine function. Moreover, its participation in the efficacy of anticancer treatments has been well described. For instance, the involvement of the breast microbiota in breast cancer (BC) development and progression has gained ground in the past several years. In this review, we report and discuss new findings on the impact of the gut and breast microbiota on BC, focusing on the HER2+ BC subtype, and the possibility of defining microbial signatures that are associated with disease aggressiveness, treatment response, and therapy toxicity. We also discuss novel insights into the mechanisms through which microorganism-host interactions occur and the possibility of microbiota editing in the prevention and treatment optimization of BC.

## Introduction

The study of the human microbiota is emerging as notable field in breast cancer (BC) research, because it is involved in many aspects of tumor biology (e.g., immune system regulation, oncogenic signaling, hormone availability, and drug metabolism) that contribute to cancer development, growth, and treatment response. Numerous studies that support the key role of commensal microorganisms in cancer development and progression have led to inclusion of the microbiota as a hallmark of various cancers (1).

The microbiota comprises all of the communities of commensal, symbiotic, and pathogenic microorganisms, including bacteria, fungi, archaea, and viruses, that colonize the gastrointestinal tract and other areas of the body (2).

To further complicate this scenario, thanks to the advance in DNA and RNA sequencing technologies, there is emerging evidence that bacteria are present, not only in the gut, but also in normal and tumor breast tissue [as reviewed extensively in (3)].

Here, we discuss new findings that link the gut and breast microbiota to BC, focusing on HER2+ BC and the consequences of this relationship on the clinical management of patients. Although several HER2-targeted treatments are available for patients with early and advanced HER2+ BCs (4) and although their use in the clinical setting has significantly improved patient outcomes, there remains considerable room for optimizing treatment strategies (5) for example by considering individual other than tumor characteristics (e.g., microbiota composition).

## Role of commensal bacteria in BC growth and progression

### Intestinal Microbiota and BC

Among human symbiotic microbial populations, the bacteria that reside in the gut have been studied and characterized most extensively. Bacteroidetes, Firmicutes, Actinobacteria, Proteobacteria, and Verrucomicrobia are the dominant phyla that inhabit the gut, with their specific distributions varying along the gastrointestinal tract, depending on competition for similar environmental conditions and nutrients (6). The gut microbiota has important physiological functions, including digestion, training of host immunity, regulation of gut endocrine function, modulation of neurological signaling, and the metabolism of xenobiotics (7). Given the complex crosstalk between intestinal microbes and their hosts, the gut microbiota is also being examined in relation to tumor development and progression.

The number of studies on the differences between the gut microbiota of patients and healthy women has been increasing (8–12), and despite the discrepancies in identified microbial taxa between them, most studies have reported reduced α-diversity in the gut microbiota of women with BC (8, 9, 12, 13). Notably, Zitvogel and colleagues showed that the fecal microbiota composition discriminates groups of patients by tumor size (< or >pT1), grade (G1 and 2 vs G3), axillary node involvement (N- vs N+), and TNM stage (stage I vs stage II/III) (14). Higher levels of bacteria, as with *Eubacterium* genera (*E. rectale, E. eligens*), *Akkermansia muciniphila, Actinobacteria* classes (*Bifidobacterium Longum, Collinsella aerofaciens)*, and *Alistipes shahii*, were associated with stage I or N negative status in BC patients, and their overrepresentation in mice is associated with slower tumor growth compared with overrepresentation of bacteria species such as *Bacteroides uniformis*, *Bacteroides xylanivolvens* and *Bacteroides intestinalis*, that reportedly associated with worse patient outcome.

The authors of this study highlighted the relevance of considering bacterial strain levels and bacterial function in discriminating patients according to their prognosis. In this cohort of patients, the expression of specific functional pathways, such as L-arginine and adenosine ribonucleotides, and the dominance of pathways like 2-oxoglutarate:ferredoxin oxidoreduction and adenine‐adenosine salvage were associated with a favorable BC prognosis, whereas biosynthesis of lipids, thiamine diphosphate, pyridoxal-5 phosphate, L-threonine, and degradation of L-histidine correlated with a poor outcome (14).

Similarly, differences in the metabolic pathways of the gut microbiome between BC and control cases were found by Yang et al. (13), strengthening the relevance of bacterial functions in the relationship between the microbial ecosystem and BC.

The use of antibiotics induces considerable changes in the intestinal microbial ecosystem, and their impact on cancer is now under investigation. An association between antibiotic use and cancer development has been reported: countries with a high consumption of antibiotics experience a higher incidence of various types of cancer (e.g., colorectal, lung, melanoma, breast, uterus, and bladder), as described by Ternàk et al. (15). Moreover, in preclinical studies, antibiotics accelerate tumor growth in various BC mouse models (16, 17), and they are also associated with early inflammation in the mammary gland, coupled with significant myeloid cell infiltration and enhanced fibrosis in a model of hormone receptor-positive BC (18).

Antibiotics use has also been associated, in a case-control study (19), to higher incidence of male BC cancer, a rare disease in men but that represents almost 1% of all BC diagnoses (20). Although there are no studies specifically aimed at investigating the gut microbiota role in male BC, commensal bacteria potentially affect BC regardless of gender, as life style, estrogen levels, inflammation and epigenetic factors represent important risks factors for BC even in male population. Indeed, intestinal microorganisms can affect BC development and progression through several mechanisms, including direct carcinogenesis (i); estrogen-dependent mechanisms (ii), such as the regulation of estrogen metabolism; estrogen-independent mechanisms including the production of microbial metabolites (iii) and regulation of the immune system (iv).

- The only bacteria that has been currently identified as a direct carcinogen in humans is *Helicobacter pylori*, which causes stomach adenocarcinomas directly (21). Nonetheless, experimental evidence highlights the cancer-initiating potential of other bacteria. These “oncomicrobes” induce cancer by genotoxic-mediated mutagenesis through toxins and virulence factors (22–28). With regard to BC, Parida et al. (29) recently showed that gut colonization with enterotoxigenic *Bacteroides fragilis* (ETBF), which secretes *B. fragilis* toxin (BFT), affects epithelial hyperplasia in the mammary gland and that *in vitro* treatment of BC MCF7 cells with BFT before injection in mice significantly increases tumor growth rate and the development of metastases through the β-catenin and Notch1 axis (29).
- The gut microbiota influences estrogen metabolism, with consequences on the well-known contribution of estrogens to the development of hormone-dependent BC. The expression of β-glucuronidase (BGUS) enzymes in bacteria, such as *Escherichia* and *Shigella*, which belong to the phylum Proteobacteria, allows sexual hormone reabsorption *via* the enterohepatic pathway, consequently increasing the levels of circulating estrogens and thus impacting the growth of BC (30, 31).
- The link between BC and the gut microbiota extends beyond estrogen-dependent pathways, in fact a diverse microbial composition and lower bacterial diversity have been observed in postmenopausal women with biopsy-proven BC compared with normal mammography, regardless of the levels of systemic estrogens (8). Among the estrogen-independent pathways that affect BC, bacteria metabolites that are derived from fiber fermentation, bile acid (BA) metabolism, lipid metabolism, and cholesterol metabolism/elimination interfere directly or indirectly with tumor cell proliferation and differentiation (32, 33). A widely known mechanism by which gut microbiota influences cancer growth is the production of short-chain fatty acids (SCFAs), such as acetate, butyrate, and propionate, which are the principal metabolites that are derived from the gut microbial fermentation of insoluble dietary fiber and are important metabolites for the maintenance of intestinal homeostasis, modulating various aspects of intestinal epithelial cells and leukocytes. They are usually taken up as a source of energy by colonocytes but also act as regulators in cells through the direct activation of G-protein-coupled receptors (GPRs) and the inhibition of histone deacetylases (HDACs) [reviewed in (34)].
  Although SCFAs inhibitory function in the development of colorectal cancer (CRC) is established (35), their role in BC is poorly understood. Overall, there is concordance in their antitumor properties as inductors of apoptosis in BC cells *in vitro* (36–39). For instance, propionate inhibits tumor growth, the epithelial-to-mesenchymal transition (EMT), and induces apoptosis in BC cells by binding to GPR43 and GPR41 receptors (39), whereas sodium butyrate induces dose-dependent inhibition of BC cell proliferation and apoptosis (40, 41).
  Similar to SCFAs, cadaverine, another bacterial metabolite that is derived from the decarboxylation of lysine and arginine, negatively affects proliferation, cellular migration/invasion, and EMT of the 4T1 BC cell line by binding trace amine-associated receptor-1 (TAAR1) (42).
  BAs are synthesized from cholesterol in the liver and are released into the small intestine to enhance the digestion and absorption of lipids and fat-soluble vitamins. Their reabsorption occurs in the ileum and colon, and only a small proportion is secreted to feces. Bacteria in the gastrointestinal tract express bile salt hydrolase enzymes and initiate BA metabolism by deconjugating the glycine or taurine from the sterol core of primary BAs (i.e., cholic acid and chenodeoxycholic acid). This mechanism prevents their reabsorption in the ileum and promotes their entrance into the large intestine, where bacteria mediate their conversion to secondary BAs [e.g., deoxycholic acid (DCA) and lithocholic acid (LCA)] (43).
  DCA results from the conversion of cholic acids to DCA *via* 7α-dehydroxylation and promotes metastases of BC tumors that have been grafted into the mouse fat pad by elevating Flk-1, a receptor for vascular endothelial growth factor, and decreasing ceramide-mediated apoptosis of BC cells (44). *In vitro* studies have shown that DCA salt has concentration-dependent effects on MCF7 BC cells, promoting cell proliferation at physiological levels through the induction of AKT phosphorylation and cyclin D1 expression and being cytotoxic at supraphysiological concentrations by inducing apoptosis (45).
  LCA is derived from the transformation of chenodeoxycholic acid and ursodeoxycholic acid through the dehydroxylation by anaerobic bacteria (primarily *Clostridiales*) and inhibits the EMT and metastasis by promoting antitumor immunity and changes in cell metabolism (33).
  Secondary BAs, like DCA and LCA, might impact tumor development and progression due to their immunosuppressive properties. In fact, DCA suppresses immune activation in models of chronic inflammation by binding to farnesoid X receptor (FXR), a BA nuclear receptor, and TGR5 (Gpbar1), a G protein-coupled BA receptor on macrophages and monocytes. Thus, while DCA confers relief under pathological conditions, such as colitis (46) and obesity-induced diabetes (47), in tumors, it might favor the establishment of a protumorigenic microenvironment. Similarly, LCA controls adaptive immunity by impeding Th1 cell activation and inhibiting interferon gamma (IFNγ) and tumor necrosis factor alfa (TNFα) release through vitamin D receptor, which is involved in BA signaling (48).
- The regulation of inflammation is another mechanism that links the gut microbiota to tumor growth (49). In fact, studies have highlighted the influence of the intestinal ecosystem on tumor immune infiltration, with its consequent impact on tumor growth (17, 18). The major function of the microbiota in the tumor immune microenvironment was elegantly studied by Lam and colleagues (50) in various tumor models, including BC, showing that the presence or absence of gut commensal bacteria discriminates between an antitumorigenic or protumorigenic immune microenvironment. Stimuli from microbiota (e.g., the microbial metabolite c-di-AMP) reprogrammed mononuclear phagocytes in the tumor into immunostimulatory monocytes and dendritic cells (DCs), which, by releasing type I IFN, promoted macrophage polarization toward an antitumor phenotype and stimulated crosstalk between natural killer (NK) cells and DCs (50). This cascade was halted in germ-free mice, which experienced differentiation of monocytes into protumor macrophages.

### Breast Microbiota and BC

Although the breast and its milk were initially thought to be sterile, several studies have demonstrated that microorganisms reside in the mammary gland, as reviewed in (51). Bacteria colonize the breast through several routes. They can derive from the skin and gain access to the mammary gland through the nipple (52, 53), translocate from the intestine (54), or reach the breast by being internalized in macrophages (55).

Normal breast tissue has a unique bacterial pattern compared with other body sites, consisting of Proteobacteria, Firmicutes, Actinobacteria, and Bacteroidetes (56–58), and similar to the gut, indications suggest that its composition is influenced by lifestyle (54) and ethnicity (59).

The diverse microbiota of breast tumors compared with their normal counterparts are likely to be implicated in BC development and progression. An analysis of the largest cohort of tumor microbiomes (including 1526 tumors from 7 types: lung, ovary, pancreas, melanoma, bone, brain, and breast) confirmed that BC has a rich and diverse microbiome and demonstrated that live bacteria exist in cancer cells and immune cells in the tumor microenvironment (55). In addition, a recent study demonstrated that tumor-resident intracellular microbiota contributes to cancer aggressiveness and to metastatic colonization by participating to the reorganization of actin cytoskeleton of tumor cells enhancing their resistance to fluid shear stress upon the entrance in the systemic circulation (60).

Compared with normal adjacent tissue, BC tissue has a significantly lower bacterial load and a more diverse bacterial composition, harboring more Proteobacteria and Firmicutes and less Actinobacteria (61, 62). These results are consistent with RNA sequencing analysis from The Cancer Genome Atlas (TCGA) (63) (using 668 BC tissues and 72 noncancerous adjacent tissues) by Thompson et al. (56), who found an increase in Proteobacteria in tumor tissues and Actinobacteria in noncancerous adjacent tissues. Further, other studies have reported that *Escherichia coli* and *Bacillus cereus* are more abundant in BC tissues than normal breast tissues (64) and genera, such as *Fusobacterium*, *Atopobium*, *Gluconacetobacter*, *Hydrogenophaga*, and *Lactobacillus*, correlated with malignancy (65). Notably, differences emerged even from an analysis of bacterial 16S rRNA sequences between normal tumor-adjacent tissue in BC women and the breast tissue of healthy volunteers, with higher relative levels of *Bacillus, Enterobacteriaceae*, and *Staphylococcus* seen in the normal tumor-adjacent tissues (66).

The content of breast microbiota might have value in discriminating patients who are at higher risk of regional recurrence, as shown by Kim et al. (67). Of note, *Porphyromonas*, *Lacibacter*, *Ezakiella*, and *Fusobacterium* were more abundant in higher-stage versus lower-stage tumors. Lymphovascular invasion was positively associated with *Lactobacillus* and correlated negatively with *Alkanindiges*, whereas node-positive status was linked to *Acinetobacter* and *Bacteroides* but negatively associated with *Achromobacter* (68). BC tissue from patients with recurrence is characterized by higher levels of *Enterococcus*, *Cutibacterium*, and bacteria that express genes that are involved in the pentose-glucoronate interconversion pathways. The possibility that the microbiota function in BC tumorigenesis is underpinned by the association of specific bacteria (e.g., *Haemophilus influenzae* and *Listeria fleischmannii*) with genes that are expressed by tumor cells, such as those that are involved in EMT, and mediate the G2-M DNA damage checkpoint, E2F transcription, and mitotic spindle assembly pathways (56). Moreover, the ability of *E. coli* (a member of the *Enterobacteriaceae* family) and *Staphylococcus epidermidis* to induce DNA double-strand breaks in HeLa cells supports their direct involvement in tumor onset (66). In particular, *E. coli*, when coupled with other molecular errors in breast tissue, can promote BC through colibactin, a genotoxin that causes double-strand DNA breaks, as has been seen in colorectal cancer (64). Protumorigenic activity has also been described for *Fusobacterium nucleatum*, an oral bacterium that has been implicated in periodontal disease that reaches the colon through the bloodstream. *F. nucleatum* is generally associated with a poor prognosis in colon cancer and was recently identified in BC samples (69). In experimental models, when injected intravenously in tumor-bearing mice, *F. nucleatum* specifically colonizes mammary tumors, promoting growth and metastatic progression and reducing tumor-infiltrating T cells (69).

Breast tissue bacteria, albeit at low abundance, may have also the potential to influence the local immune microenvironment: Tzeng and colleagues found, in patients, an association between specific bacteria and local immune infiltrates, based on the expression of immune genes. In healthy controls and tumor tissue, *Acinetobacter* correlated positively with CD8+ T cell levels. In tumor tissue, *Methylibium*, *Pelomonas*, and *Propionibacterium* were significantly associated with immune genes, with *Methylibium* correlating negatively with ICOS and TBX21 expression and T-cell abundance and *Propionibacterium* negatively associating with IP-10 and MIP-1B, two effector molecules that are produced downstream of TLR activation (68).

## Commensal bacteria and HER2+ BC

### Association Between Microbiota Composition and HER2+ BC Subtype

Novel insights into the association between the microbial signature and BC subtypes have been emerging. In this section we focus on microbiota composition found to be associated with the expression of HER2+ receptor (**Table 1**). With regard to the gut microbiota, fecal samples from women with HER2+ BCs are characterized by lower α-diversity and lower levels of certain genera of Firmicutes (i.e., *Clostridium, Blautia, Coprococcus, Ruminococcus, SMB53 genus*), whereas they have more *Bacteroidetes* compared with HER2- patients (70), suggesting that specific gut microbial compositions may represent a risk factor for this tumor subtype. Further evidence was presented by Yang and colleagues (13), who, in contrast to Wu et al., found a higher proportion of bacterial taxa that belonged to Firmicutes (*Megasphaera*, *Lachnospiraceae*, *Flavonifractor*, and *Eubacterium*), Bacteroidetes (*Barnesiellaceae*, and *Alloprevotella*), Proteobacteria (*Moraxellaceae*, *Acinetobacter*, *Pseudomonadales*, and *Burkholderiaceae*), and Actinobacteria (*Enorma*) in the gut of HER2+ BC patients.

**Table 1:** Bacterial signature in HER2+ BC subtype. BC, breast cancer; FFPE, formalin-fixed paraffin-embedded.

Study	Sample type	Cohort	Methodology	Microbiota in HER2+BC compared to HER2-BC
**Wu et al.** (2020) (70)	Fecal samples	HER2+ n=12 HER2-n=25	16S rRNA gene sequencing	Lower α-diversity Bacteroidetes *(Alistipes)* Firmicutes *(Enterococcus*, *Acidaminococcus).* Bacteroidetes *(Rikenellaceae)*, Euryarchaeto *(Methanobrevibacter)*, Firmicutes *(Christensenellaceae*, *Turicibacter, Clostridium, SMB53*, *Blautia, Coprococcus, Ruminococcus)* Proteobacteria *(Desulfovibrio)*
**Yang et al.** (2021) (13)	Fecal samples	HER2+ n=37 HER2-n=45	16S rRNA gene sequencing	Firmicutes (i.e., *Megasphaera*, *Lachnospiraceae*,*Flavonifractor*, and *Eubacterium*), Bacteroidetes (i.e., *Barnesiellaceae* and *Alloprevotella*) Proteobacteria (i.e., *Moraxellaceae*, *Acinetobacter*, *Pseudomonadales* and *Burkholderiaceae*) Actinobacteria (i.e., *Enorma*)
**Wang et al.** (2017) (71)	BC tissue frozen	HER2+ n=8 HER2-n=36	16S rRNA gene sequencing	No significant differences
**Smith et al.** (2019) (59)	BC tissue frozen	HER2+ n=6 HER2-n=51	16S rRNA gene sequencing	Thermi Verrucomicrobia *(i.e., Akkermansia)*
**Nejman et al.** (2020) (55)	BC tissue FFPE	HER2+ n=61 HER2-n=247	16S rRNA gene sequencing	Firmicutes *(Granulicatella:US31)* Bacteroidetes *(Dyadobacter)*
**Tzeng. et al.** (2021) (68)	BC tissue frozen	HER2+ n=15 HER2-n=206	16S rRNA gene sequencing	Firmicutes *(Filibacter* and *Anaerostipes)*, Bacteroidetes *(Cloacibacterium* and *Alloprevotella)* Proteobacteria *(PRD01a011B*, *Stakelama Blastomonas)*
**Hadzega et al.** (2021) (58)	BC tissue frozen	HER2+ n=4 HER2-n=14	RNA-sequencing	Proteobacteria (i.e. *Burkholderiales* and *Helicobacter pylori)*

Most studies that have reported differences in microbe composition between BC subtypes have focused on the breast tissue microbiota. For instance, Wang et al. identified significant clustering in an unweighted UniFrac analysis by HER2 amplification status (71). Among the four major BC subtypes, women with HER2+ tumors were enriched for the genera *Akkermansia* (phylum *Verrucomicrobia*) and *Thermi*, whereas triple negative breast cancer (TNBC) had the highest levels of *Euryarchaeota, Cyanobacteria*, and Firmicutes. The phyla Tenericutes, Proteobacteria, and Planctomycetes were greater in luminal subtypes (59). A comparison of HER2+ (n=61) and HER2- (n=247) BCs by the Straussman group revealed that BCs that overexpressed HER2 were enriched for *Granulicatella_US31* (Firmicutes) and *Dyadobacter* (Bacteroidetes) (55).

A similar analysis in a series of 221 BCs using 16S rRNA gene sequencing noted significantly higher levels of 7 genera of Firmicutes (*Filibacter* and *Anaerostipes)*, Bacteroidetes *(Cloacibacterium* and *Alloprevotella*), and Proteobacteria (*PRD01a011B*, *Stakelama*, and *Blastomonas*) in HER2+ versus HER2- tumors (68). Using transcriptomic RNA-Seq data, Hadzega et al. observed the overrepresentation of Proteobacteria (*Burkholderiales* and *Helicobacter pylori)* in HER2+ BCs compared with tumors with other molecular subtypes (58).

Although these studies strongly suggest the existence of a BC subtype-specific microbial composition, we are still far from defining a consensus microbial signature for HER2+ BCs because there is no agreement with regard to the bacterial taxa that are associated with the HER2+ subtype or their levels in the gut/BC tissue.

### Microbiota and Response to HER2+ BC Treatment

**Figure 1:**
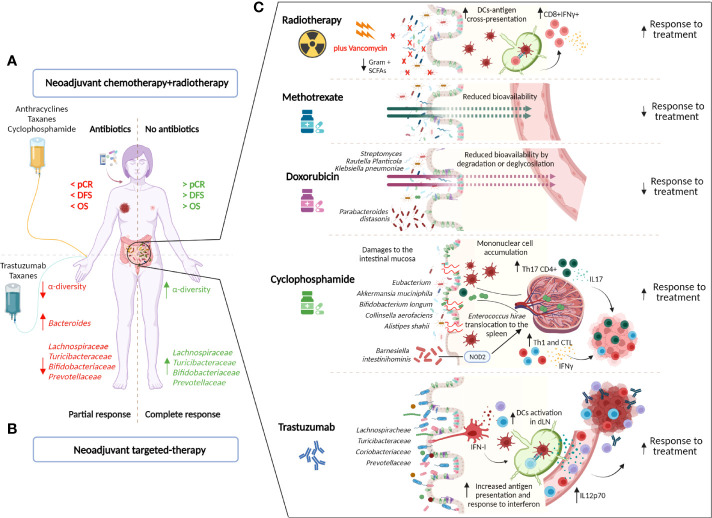
Impact of the gut microbiota on the treatment of HER2+ BC patients. Current HER2+ BC treatments comprise radiotherapy (RT), chemotherapy (anthracyclines, taxanes, cyclophosphamide (CTX), methotrexate, and 5-fluorouracil), and anti-HER2 agents (trastuzumab). The gut microbiota can affect treatment efficacy by directly influencing drug metabolism or by shaping the host’s immune response to the treatment. In patients, the use of antibiotics near the neoadjuvant therapy negatively affects the response to treatment and is associated with lower disease-free (DFS) and overall (OS) survival (72). **(A)**. Moreover, an intestinal microbiota that is low in bacteria that belong to the taxonomic families *Lachnospiraceae*, *Prevotellaceae*, *Actinobacteria* (*Bifidobacteriaceae*), and *Turicibacteriaceae* but enriched in *Bacteroides* is associated with the response to trastuzumab-containing neoadjuvant chemotherapy (73). **(B)**. Several mechanisms have been proposed to explain the influence of gut microbes on anticancer treatment. **(C)**. Radiotherapy induces DNA damage in cancerous cells and causes immunogenic cell death, eliciting adaptive antitumor immunity, due to tumor antigen cross presentation by dendritic cells (DCs) to cytotoxic CD8+ T cells. The depletion of vancomycin-sensitive gram+ bacteria enhances DC antigen presentation, improving RT efficacy (74). Methotrexate and doxorubicin can be converted into downstream metabolites, reducing their bioavailability (75, 76). In particular, *Streptomyces* and *Raoultella >planticola* inactivate doxorubicin by deglycosylation, whereas *Klebsiella pneumoniae* reduces its bioavailability by degradation (76). Moreover, gut colonization with *Parabacteroides distasonis* is associated with compromised anticancer efficacy (77). CTX damages the gut mucosa, rendering the gut leaky and allowing bacteria to translocate to secondary lymphoid organs (e.g., spleen). The translocation of *Enterococcus hirae* stimulates a Th17 immune response (77), whereas the accumulation of *Barnesiella intestinihominis* stimulates a Th1 response through a NOD2-dependent pathway (78). In this context, these two microbes participate in CTX efficacy by favoring the accumulation of cytotoxic cells in the tumor burden. Moreover, gut colonization with *Eubacterium rectale, Eubacterium eligens, Akkermansia muciniphila, Bifidobacterium longum, Collinsella aerofaciens*, and *Alistipes shahii* favors CTX efficacy in mice (14). With regard to trastuzumab, the maintenance of a healthy intestinal ecosystem with higher levels of *Lachnospiraceae*, *Turicibacteriaceae*, *Coriobactriaceae*, and *Prevotellaceae* compared with *Bactoridales*, *Proteobacteria*, and *Verrucomicrobia* maintains proper immune tone in the steady state, which leads to antigen processing and presentation at the ileum level and the activation of an inflammatory response and the type I IFN pathway, which induces commensal bacteria to instruct mononuclear phagocytes, such as DCs, and, on trastuzumab treatment, increases IL12p70 levels to activate NK and T cells against tumors (73) (created by Biorender).

Current HER2+ BC treatments comprise surgery, radiotherapy, and chemotherapy (anthracylines, taxanes, CTX, methotrexate, and 5-fluorouracil), coadministered with various anti-HER2 compounds (trastuzumab, pertuzumab, drug-conjugated trastuzumab (ADCs), and tyrosine kinase inhibitors). Bacteria can condition the bioavailability, toxicity, and efficacy of chemotherapeutic drugs with possible effects on patient prognosis (**Figure 1**). It is also important to note that patients outcome could be affected by the alteration of the microbiota composition following chemotherapeutic treatment. In fact, anthracyclines can be bacteriostatic to *Acinetobacter* species (79), whereas gemcitabine has bactericidal properties (80, 81);. CTX damages the gut mucosa, rendering the gut leaky and allowing bacteria to enter the bloodstream (82). Methotrexate induces changes in the diversity and abundance of bacteria that are associated with chemotherapy-induced diarrhea (83). In BC patients, neoadjuvant treatment with anthracyclines, CTX and taxanes, shifts the breast tumor microbiome compared with tumors from untreated patients toward a lower α-diversity and an increase in *Pseudomonas* spp, the metabolites of which can increase chemotherapeutic efficacy (84). Also, selective estrogen receptor modulators used in combination with anti-HER2 treatment in patients with HER2+ and ER+ BC, such as tamoxifen and raloxifene, can change the composition of the gut microbiome (85–89).

On the other side, commensal bacteria regulate anticancer drug efficacy in different ways. Methotrexate bioavailability is reduced by its conversion into a downstream metabolite by bacteria (75) whereas doxorubicin can be inactivated by different bacteria through deglycosylation (*Streptomyces* WAC04685 and Raoutella *planticola*) or degradation (*Klebsiella pneumoniae*) (76).

The microbiota is also critical in mediating chemotherapeutic toxicity. For instance, doxorubicin-induced intestinal injury and cardiac dysfunction are associated with an imbalance in the microbiome (90, 91), whereas treatment with taxanes and antimicrotubule agents decrease the abundance of *A. muciniphila* and consequently disrupt gut barrier integrity, resulting in enhanced neuropathy and altered brain function (92). Also, the toxicity of methotrexate is influenced by the gut microbiota’s immunomodulatory properties: gut microbes alleviate chemotherapy-induced small intestinal injury through the regulation of the multidrug transporter ABCB1/MDR1 p-gp by TLR2 signaling (93). Based on this evidence, several studies have investigated the possibility of exploiting nutraceutical interventions to ameliorate the adverse reaction of taxanes and, in general, of chemotherapeutic drugs (94, 95).

In addition to the regulation of drug metabolism and toxicity, the gut microbiota influences their efficacy directly or by modulating the immune response. Doxorubicin activity likely depends on the absence of specific microbes in the gut, in fact, it retains the therapeutic potential in microbiota-depleted mice, but gut recolonization with *Parabacteroides distasonis* compromises its anticancer efficacy (77). The antitumor activity of CTX is mediated by the translocation of gram-positive bacteria into intestinal secondary lymphoid organs, leading to a Th17 and Th1 immune response (77). In this context, *Enterococcus hirae and Barnesiella intestinihominis* have been demonstrated to participate in the efficacy of CTX by favoring the accumulation of cytotoxic cells in the tumor burden (78). Notably, a cross-reaction between commensal-specific memory T cells and tumor-associated antigens appears to form the basis of the gut microbiota’s influence on the efficacy of CTX (96). Further, Zitvogel and colleagues reported the direct involvement of specific bacteria (*E. rectale, E. eligens, A. muciniphila, B. longum, C. aerofaciens*, and *A. shahii*) in CTX antitumor efficacy in BC patients (14).

Antibiotics severely disrupt intestinal ecosystem, their impact on neoadjuvant treatment in BC was examined in the study by Zhang et al. (72), in which 120 BC patients were analyzed. Patients who received antibiotic treatment within 30 days after initiation of neoadjuvant therapy were compared with control patients who avoided antibiotic medication. Overall, the pathological complete response rate of the control group was significantly higher than that of the antibiotic-treated group, and there was a strong link between antibiotic consumption and worse DFS and OS. Notably, among HER2+ BC patients (n=46) who received docetaxel, anthracycline and CTX, the use of antibiotics (n=19 vs n=23 patients in the control group) was significantly associated with lower efficacy of the neoadjuvant therapy and worse DFS and OS (72). Although limited by its retrospective design and the inclusion of a small number of cases, this study calls clinicians’ attention to antibiotic use during chemotherapy and targeted therapy.

Radiotherapy (RT) is another widely used antitumor treatment that is usually combined with chemotherapy. In addition to damaging the DNA of cancerous cells, RT may affect immunogenic cell death and elicit adaptive antitumor immunity through the cross-presentation of tumor-associated antigens to CD8+ cytotoxic T lymphocytes by APCs (97), generating an immune response that impacts distant non-irradiated tumor foci, known as the abscopal effect (98). Although there are no data specifically related to BC patients, the gut microbiota modulates the antitumor immune response following RT distal to the gut. In particular, the depletion of vancomycin-sensitive bacteria enhances DC antigen presentation, improving the antitumor activity of RT (74). Thus, to optimize the cure and prognosis of BC, further examination of the gut and breast microbial components and their interaction with tumor cells and therapies can not be ignored.

### Microbiota and Efficacy of Anti-HER2 Targeted Therapy

The introduction in clinical setting of anti-HER2 targeted therapy significantly improved the prognosis of this aggressive tumor subtype. Several HER2-targeting agents are available for the treatment of early, advanced and metastatic HER2+BC, and include monoclonal antibodies (trastuzumab, pertuzumab), tyrosine kinase inhibitors (lapatinib, neratinib), and ADCs (trastuzumab-trastuzumab-TDM1 and trastuzumab deruxtecan).

Despite the improvement in the clinical outcome of HER2+ BC patients, high heterogeneity characterizes the response to targeting agents [reviewed in (4, 99)] and patients can experience disease recurrence after curative intent and disease progression in the metastatic setting. The host immune response plays a key role in the activity of anti-HER2 monoclonal antibodies. In particular, while trastuzumab binds HER2, in addition to preventing HER2 receptor dimerization and blocking downstream signaling, the Fc fragment of the antibody interacts with Fc receptor expressed on innate immune effector cells, such as NK cells, macrophages, neutrophils, and γδ T cells, and activates antibody-dependent cellular cytotoxicity or phagocytosis. This cytotoxic activity increases the availability of tumor antigens in the tumor immune microenvironment, favoring their processing and presentation by antigen-presenting cells (APCs). Thus, the interaction between trastuzumab and the innate immune system facilitates the development of tumor-specific T cell immunity. NK cells prime DCs, increasing tumor antigen presentation to cytotoxic CD8+ T cells and the polarization of CD4+ T cells toward an antitumor Th1 phenotype. Yet, trastuzumab-dependent NK cell activation leads to cytokine secretion, contributing to the recruitment and functional polarization of myeloid cells and T cells (100). Thanks to the interplay between microbes and hosts immune systems the microbiota is emerging as a relevant area of focus in the management of cancer patients (101).

Recently, our group studied the impact of the intestinal microbiome on the immune-mediated antitumor efficacy of trastuzumab (73). In preclinical models of HER2+ BC, modulation of the intestinal microbiota by antibiotics decreased the antigen processing and presentation pathways at the ileum level and diminished the inflammatory response and type I IFN pathway, preventing commensal bacteria from instructing mononuclear phagocytes, such as DCs, to maintain the proper immune tone in the steady state. This disruption resulted in low circulating levels of IL12p70, a cytokine that is produced mainly by DCs and is crucial for activating NK and T cells, and in impaired NK and tumor-infiltrating T lymphocyte activity on administration of trastuzumab.

Notably, in HER2+ BC patients who received neoadjuvant treatment (ie, adriamycin plus CTX and taxanes plus trastuzumab), those who were nonresponsive had lower bacteria α-diversity and more *Bacteroides* than patients who achieved a pathological complete response. In particular, similar to mice under antibiotic treatment, nonresponsive women had low levels of members of the taxonomic families *Lachnospiraceae*, *Prevotellaceae*, *Actinobacteria* (*Bifidobacteriaceae*), *Turicibacteriaceae*, and *Desulfovibrio* in the gut. Moreover, fecal microbiota β-diversity, which segregated patients according to response, correlated positively with tumor immune features that were related to the activation of DCs and with the production of IL12p70. The direct link between the patient’s gut microbiota and the response to trastuzumab was confirmed by fecal microbiota transplantation from patients into recipient mice. This proof of the existence of a gut microbiota/immune-mediated trastuzumab axis should encourage future studies to focus on these microorganisms and their products in regulating the efficacy of HER2+ BC treatment.

## Strategies to modulate microbiota in BC

The above-described role of gut and mammary microbiota in the progression and response to therapy of BCs provides a strong foundation for pursuing approaches directed against key constituents of the cancer microbiota or modulating gut microbiota as complementary strategy to reduce anti-cancer therapy-related toxicity, improve response to treatment and ultimately prognosis in BC patients.

Currently, several options can be used to alter the composition of the microbiota, including (i) fecal transplants, which are under investigation for refractory immunotherapy (102–104), (ii) the transfer of defined bacterial consortia (105) or single bacteria isolates (78, 106), (iii) the administration of prebiotics or dietary interventions to shift exiting commensal communities (107), and (iv) the depletion of community members, ranging from broad to selective depletion through antibiotics (74, 108).

Compared to cancers treated with immunotherapy (e.g. melanoma, renal cell carcinoma), few studies are ongoing in BC patients and almost all of them are observational studies still in the first step, mainly aiming to assess the association between gut and breast microbiota, the immune system and response to therapy. Only recently three intervention trials (NCT04139993, NCT03358511, NCT03290651) are addressing the possible benefit and side effects, the systemic immunomodulatory effects of different probiotics and the associated change in breast microbiota.

**Figure 2:**
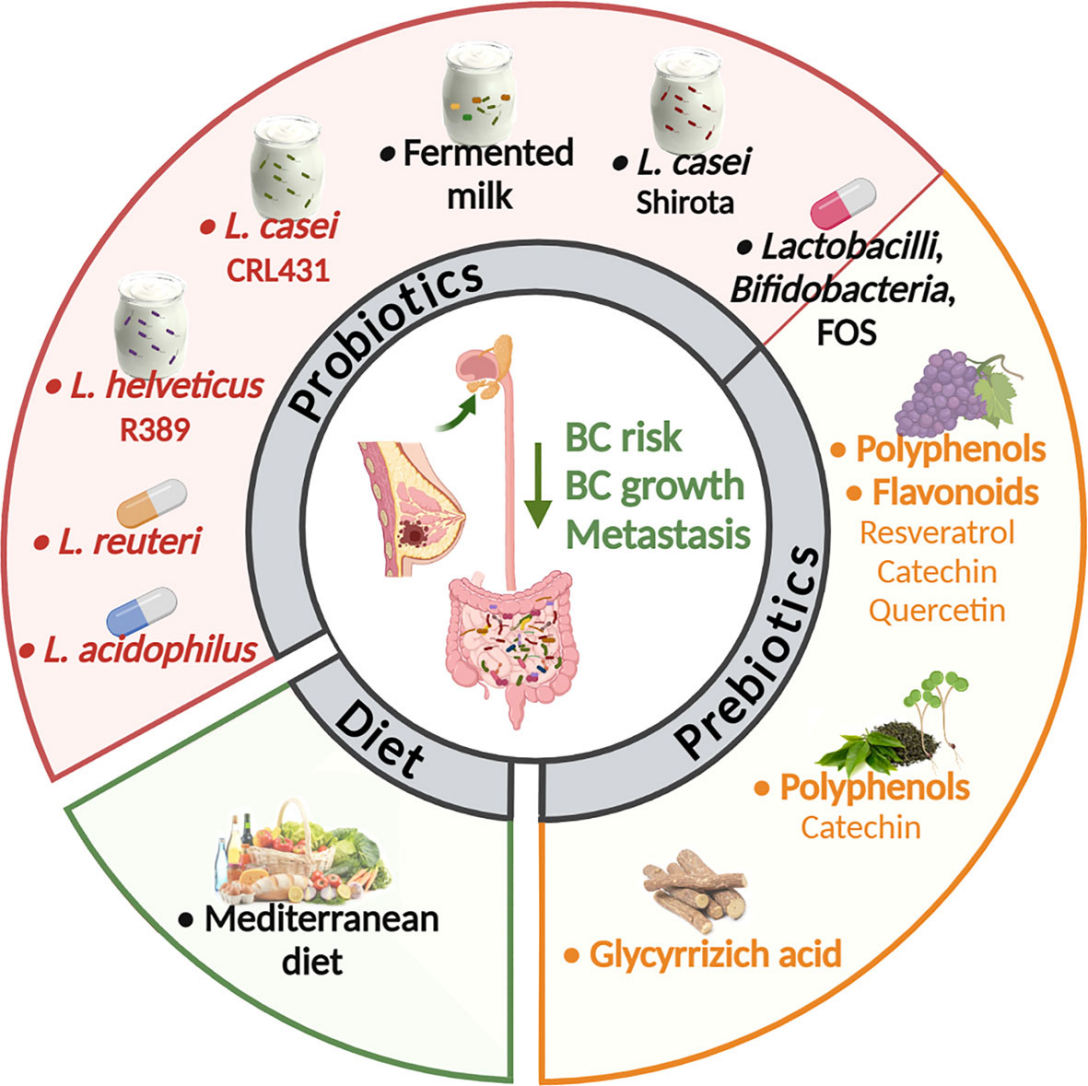
Strategies to modulate the microbiota in BC murine models and patients. Many options to alter the composition of the gut and tumor-associated microbiota are under investigation as a clinical strategy in cancer treatment and prevention. In BCs the anti-tumor activity of probiotics, prebiotics and diet was explored mainly in preclinical models. Only few studies (in black) found an association between the use of probiotics, as soy milk fermented with *Lactobacillus casei* Shirota (109) or fermented milk (110), and of a Mediterranean diet (111) with a reduced risk to develop BC in women. In another study the oral administration of different *Lactobacilli* and *Bifidobacteria* in combination with fructooligosaccharide (FOS) modified circulating risk factors, potentially reducing BC risk (112). In preclinical models, oral administration of *Lactobacillus acidophilus* or *Lactobacillus reuteri* live bacteria (113, 114) or fermented milk containing *Lactobacillus helveticus* or *Lactobacillus casei* (115, 116) inhibited mammary carcinogenesis or 4T1 BC growth. *Lactobacillus casei* also had anti-metastastic effects and diminished the capecitabine side effects (117). Also, the use of non-digestible food ingredient that promotes the growth of beneficial microorganisms in the intestines (prebiotics) was tested in preclinical models of BC. In details, oral delivery of polyphenols derived from grapes (118) or from green tea in association with broccoli sprouts (119), or the flavonoid quercetin (120), reduced primary BC growth and metastasis. Also, the glycyrrhizic acid, a phytochemical derived from licorice roots, ameliorated high fat diet-induced metastases of 4T1 BC through the modulation of gut microbiota composition (121) (created by Biorender).

On the contrary, some options of microbiota modulation have been explored in BC preclinical models (**Figure 2**). Regarding supplementation of probiotics, different studies explored the effect of lactic acid bacteria. Lakritz et al. (113) demonstrated that oral administration of *Lactobacillus reuteri* ATCC-PTA-6475 was sufficient to inhibit mammary carcinogenesis in outbred Swiss mice at increased risk of development of mammary tumors due to the feeding of a Westernized diet (high fat, low fibers), and to delay or completely prevent tumor onset in MMTV-neu mutant FVB mice, genetically predisposed to develop mammary tumors, through the induction of anti-inflammatory CD4+ CD25+ Tregs. These data are consistent with a previous study showing that administration of *Lactobacillus acidophilus* to mice with 4T1 mammary carcinomas induced a significant reduction in tumor growth by altering the cytokine production toward a Th1 profile (114). Moreover, administration of fermented milk containing *Lactobacillus helveticus* R389 decreased the growth rate of 4T1 tumors (115) impacting on cancer cell apoptosis and reducing the production of pro-inflammatory cytokines (122). Also the use of *Lactobacillus casei* CRL 431, a recognized probiotic strain in humans, to ferment milk, reduced 4T1 tumor growth stimulating the immune response against the tumor, when administered as preventive therapy or after tumor injection (116, 123). In 4T1 mouse mammary tumors, this probiotic was also demonstrated to have anti-metastastic effects and to diminish capecitabine side effects without affecting the treatment efficacy (117).

Consistent with preclinical data indicating that lactic acid bacteria could act as a great immune adjuvant for combined BC therapy, a randomized clinical trial including 76 overweight or obese postmenopausal women with a history of hormone-receptor-positive BC showed that the administration of symbiotic supplements containing *L. casei*, *L. acidophilus*, *L. rhamnosus*, *L. bulgaricus*, *B. breve*, *B. longum*, and *Streptococcus thermophilus* and fructooligosaccharide (FOS) contributed to a significant increase in adiponectin and decline of TNFα, and high-sensitivity C-reactive protein (hs-CRP) compared with placebo, suggesting beneficial effects of synbiotic supplementation on recurrence risk factors (112). Moreover, in a population-based case-control study in Japanese women it was demonstrated that regular consumption since adolescence of *L. casei Shirota*, which modulates the composition and metabolic activity of the intestinal microbiota (124), is significantly inversely associated with the incidence of BC (109). These findings explain at least partially previous evidence that the high intake of fermented milk products may lower the risk of BC in women (110).

Although in these studies the modification of the gut or the tumor microbiota by lactic acid bacteria was not analyzed, it is likely that the anti-tumor activity of these *Lactobacilli* that in part depends on the modulation of the immune system, could rely on their ability to alter the microbiota. Indeed, it is known that certain strains of these bacteria not only can modulate the local immune response in the intestine, but are also effective in modulating the systemic immune responses. A direct anti-tumor activity of these bacteria is also possible based on *in vitro* data [reviewed in (125)] and on the possibility that they can migrate to the tumor tissue. Indeed, labeled oral probiotics were identified in the mouse breast milk, suggesting that gut bacteria may be able to travel to the mammary gland (126). Likewise, microbial composition of normal mammary gland and tumor were found modulated in response to diet both in a non-human primate model and in humans (54, 127), demonstrating that oral interventions can influence microbial populations outside of the intestinal tract in distal sites such as the mammary gland. In particular, in a non-human primate model (127), consumption of Mediterranean diet, which is associated with reduced BC risk (111), led to an increased abundance of *Lactobacillus* in the mammary gland compared to western diet, whose consumption reportedly elevates BC risks (128).

Another emerging option for targeting the gut microbiome and prevent BC progression is the use of compounds able to modulate gut microbiota as polyphenols and plant-derived phytochemicals, which are included in the list of prebiotics. Oral delivery of grape polyphenols (resveratrol, catechins and quercetin) was found to decrease primary tumor growth and metastases in a mouse xenograft model created from green fluorescent protein-tagged MDA-MB-435 bone metastatic variant BC cells (118). Accordingly, quercetin dietary delivery was shown to be effective in reducing tumor number and volume also in a model of spontaneous BC (C3/SV40 Tag) (120). In another study carried out in MMTV-neu wild type FVB mice, the oral delivery of green tea polyphenols, particularly enriched in catechins, and the administration of a diet supplemented with broccoli sprouts modified gut microbial composition. In particular, it was observed an increase in *Lactobacillus* and *Lachnospiraceae* paralleled by augmented levels of important bacteria metabolites, such as SCFAs. These modifications resulted in the delay of mammary tumor growth (119). Further, a study demonstrated that glycyrrhizic acid, a plant-derived phytochemical, can ameliorate HFD-induced metastases in 4T1 mammary tumor model. This effect is mediated by alteration of gut microbiota composition and concurrent reduction of colonic lipopolysaccharide (LPS) and proportion of M1-like macrophages producing CCL2 and TNFα in the colons *via* LPS/HMGB1/NF-κB, thus leading to inhibition of myeloid-derived suppressor cells deputed to formation of pre-metastatic niches (121).

Therefore, even though the research in the field is still in its infancy, diet, which is recognized as a major shaper of the complex gut microbiota ecosystem, probiotics and prebiotics consumption may represent potential regimens to modulate BC risk and increase clinical benefit of treatments.

## Discussion

Although the definition of the molecular intrinsic properties of BC cells and their dynamic interplay with the surrounding microenvironment has led to the identification of many pathways that are involved in tumor development, aggressiveness, and the response to treatment, this knowledge has been insufficient in prognosticating the risk of disease, follow-up, and, consequently, the most adequate treatment. Thus, other factors in the cancer cell/breast tissue crosstalk oversee intersubject heterogeneity in disease outcomes. Considering the studies that have been discussed in this review, the microbes that reside in the gut or breast tissue are key planners and add relevant pieces to the BC puzzle.

However, there remain critical issues to examine in determining the exact function of commensal microorganisms in the development and treatment of BC. Whether they are alive and have a direct role in BC tumorigenesis or the response to therapy still needs to be studied in detail. Few studies have demonstrated the translocation of enteric bacteria to the breast and their active role in cancer development and progression (i.e., enterotoxigenic *B. fragilis*). Most research has been limited to 16S rRNA gene-based microbial profiling or whole shotgun metagenomics (WSM), which have yielded taxonomic characterization and the prediction of functional pathways, respectively. Further, the lack of consistency in microbial profiles between patient cohorts necessitates standardized protocols for sample collection and analyses that can be applied worldwide.

Nonetheless, the definition of individual microbial species or operational taxonomic units that are responsible for tumor development and the sensitivity or resistance to treatment should not be the ultimate goal of research. In fact, major redundancies in pathways between species (i.e., that two bacterial species can perform the same function but also vary their activity according to the substrate that is available) might explain the differences in microbial profiles that are relevant in BC and warrant a move toward considering the overall function of the microbial ecosystem as the major driver of the bacterial impact on cancer. These factors must be considered in future studies to advance existing preclinical models and metagenomic technologies and develop new platforms. Further contributing to the evolution of BC, microbiota taxa take part in the responsiveness to treatment regimens, as supported by the compelling results above, shedding light on the relevance of considering the gut and, likely, the tumor microbiota in planning drug combinations and therapy regimens.

Although it is complex and dynamic, the interplay between the immune system and tumor is instrumental for the success of HER2-targeting agents (mAbs, TKIs, and ADCs). Thus, thanks to its immunomodulatory properties, the host-microbial ecosystem may contribute to the heterogeneity in the response to anti-HER2 treatment. Thus, the identification of a specific bacterial signature that is associated with treatment efficacy and the mechanisms through which it occurs may provide the missing clues in optimizing cures for HER2+ BC patients.

Several options can be used to alter the composition of the microbiota, including (i) fecal transplants, which are being investigated for refractory immunotherapy (102–104), (ii) the transfer of defined bacterial consortia (105) or single bacteria isolates (78, 106), (iii) the administration of prebiotics or dietary interventions to shift exiting commensal communities (107), and (iv) the depletion of community members, ranging from broad to selective depletion through antibiotics (74, 108). It is therefore important for more research to be carried to investigate the best approaches which can ameliorate the management of BC.

## Conclusion

This review has discussed the relevance of considering the gut microbiota in the treatment of BC patients—in particular, HER2+ tumors, for which the immune system is being increasingly implicated as a mediator of treatment efficacy and as a predictive biomarker.

Emerging evidence indicates that bacteria are present in BCs and associate with tumor hallmarks and that their relative abundance depends on the intestinal microbial ecosystem. Thus, the next challenge will be to determine the effects that intratumor bacteria have on various phenotypes of cancer cells, the immune microenvironment, and its interactions with tumor cells and, consequently, on disease outcomes.

Knowing the favorable gut/BC microbiota status (i.e., its composition and functions) for anti-HER2 drugs’ efficacy will influence the decision over de-escalation strategies in terms of chemotherapy-free, single agents versus dual blockade, and add-on strategies, such as dual blockade plus chemotherapy and immune checkpoint inhibitors, minimizing overtreatment in patients who would benefit from single agents, such as trastuzumab.

## Author Contributions

ET, TT, and MM conceptualized the review and wrote the manuscript. MM and VA conducted the collection of the literature. ET, TT, and LS provided professional revision of the article. All authors edited the manuscript and approved the submitted version.

## Funding

This work was funded by AIRC under IG 2017 - ID. 20264 project – P.I. ET.

## Conflict of Interest

The authors declare that the research was conducted in the absence of any commercial or financial relationships that could be construed as a potential conflict of interest.

## Publisher’s Note

All claims expressed in this article are solely those of the authors and do not necessarily represent those of their affiliated organizations, or those of the publisher, the editors and the reviewers. Any product that may be evaluated in this article, or claim that may be made by its manufacturer, is not guaranteed or endorsed by the publisher.
